# Erythema Ab Igne: A Mottled Rash on the Torso

**DOI:** 10.7759/cureus.6628

**Published:** 2020-01-11

**Authors:** Kelsey M LeVault, Amit Sapra, Priyanka Bhandari, Madelyn O'Malley, Eukesh Ranjit

**Affiliations:** 1 Family Medicine, Southern Illinois University School of Medicine, Springfield, USA

**Keywords:** infrared radiation, heat, reticulate, coal stoves, chronic pain

## Abstract

Erythema ab igne (EAI) is a typical example of an environmental-induced dermatosis secondary to overexposure of a particular part of the skin to heat. Once a familiar entity in the precentral heating era, it seems to be making a comeback with prolonged usage of electronic devices close to the body surface as well as usage of alternative methods of pain relief being sought by patients. We describe a case of a 39-year-old female who presented to our clinic with a mottled reticulate rash on her back after five years of using heating pads for her chronic backache.

## Introduction

Erythema ab igne (EAI), also known as the toasted skin syndrome [1], is a skin condition seen in patients who have extended exposure to local or regional heat. It is a typical example of an environmental-induced dermatosis. It was a familiar entity before the era of central heating in western countries around the mid-19th century. It is still commonly seen in developing countries, especially in rural areas where people use warming methods close to the body surfaces during the winter seasons. In the past several decades, EAI has returned to Western countries through the phenomenon of having electronics such as laptops in prolonged contact with body surfaces [2-3]. It also seems to be increasingly reported as patients with chronic pain are utilizing heat as an alternative method of pain relief secondary to a substantial cut down on pain prescriptions by the providers [4].

## Case presentation

The patient is a 39-year-old female with a past medical history of chronic low back pain, degenerative disc disease of the lumbosacral spine, lumbar radiculopathy, migraine headaches, and hypothyroidism, who presented to our clinic to establish primary care. She stated that she had been suffering from chronic low backache since the age of 26, for which she had been using pain medication on and off. Over the past five years, the patient started using heating pads as an alternative method of pain relief.

She also stated that she had undergone physical therapy in the past without much benefit. She informed us that for over five years now, she had been using two heating pads covering her lower back. She stated that she had been using them several hours during the day, especially during the evening. She said she has also been sleeping with them covering her lower back. The electric heating pads were applied at medium to low settings most of the time.

At the time of establishing care with us in the clinic, she also presented with a rash, for the last one year, on her back corresponding to the area where she had been chronically using her heating pads. She denied any pain, itching, or any discharge from the rash or any similar rash anywhere else on the body. She also denied any known personal or family history of skin problems in the past.

**Figure 1:**
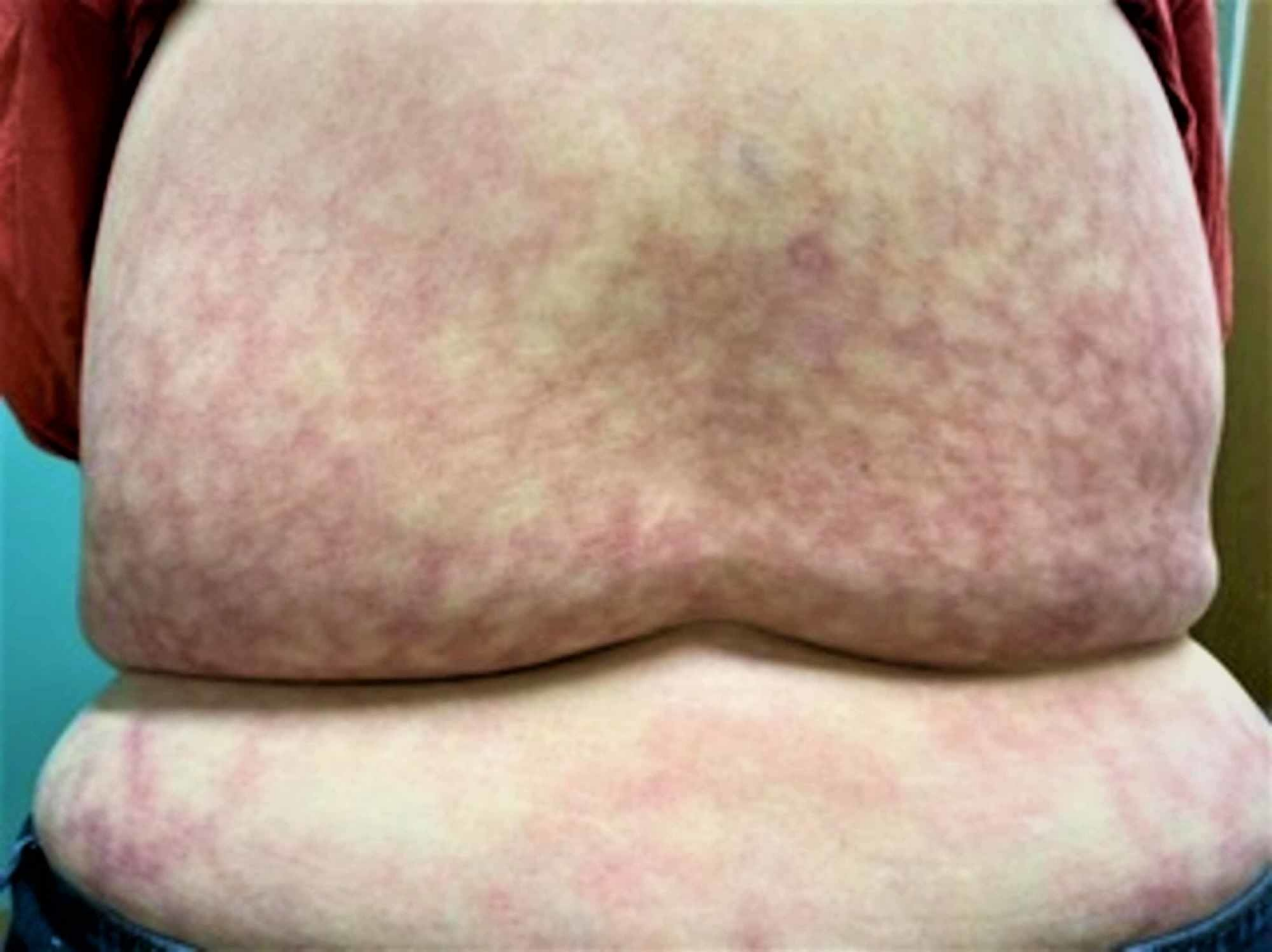
The affected area on the back corresponding to where the patient placed the heating pads showing a reticulte, hyperpigmented pattern suggestive of erythema ab igne

**Figure 2:**
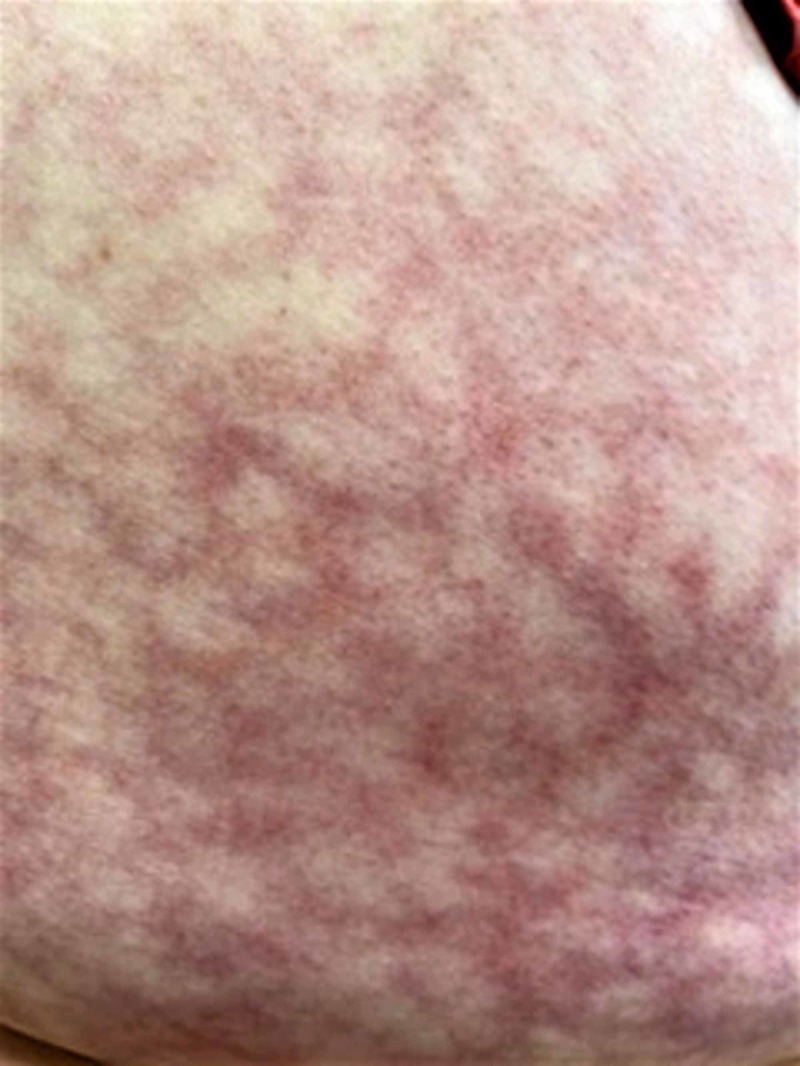
Enlarged view of the patch where the reticulate, erythemtous pattern with telangiectasias can be clearly appreciated

On examination, there was a non-blanching, violaceous, mottled, reticulate patch on her back corresponding to the lower thoracic and lumbosacral area (Figure 1). The lesion was erythematous with telangiectasias (Figure 2). The patch was not raised and nonpruritic in nature. No other skin lesions were observed anywhere else on the body. Based on the history and physical exam, a diagnosis of EAI was made. The patient was educated about the skin lesion and was advised to stop using heating pads over the affected area. She followed with us six weeks later and stated that she had stopped the use of heating pads. The physical exam was at that time still unchanged from the last visit. The patient was encouraged not to use heating pads again and was informed that it might take up to a few months for the rash to resolve.

## Discussion

EAI is a Latin word meaning “redness from fire.” This condition was first described by a German dermatologist named Abraham Buschke. He called it “hitze melanose,” which, when translated, means darkening due to heat [2].

As an environmental and occupational dermatosis, it is characterized by a local erythematous, hyperpigmented reticulated net-like pattern of the skin surface that has been in contact with the heat or infra-red radiation. Historically, it was first observed in women who worked with coal stoves, with direct exposure of heat to their legs. It had also been observed with people exposed to kerosene stoves as well as wood-burning stoves. There is abundant literature on this condition developing with the use of space heaters, heating pads, heated chairs, warm water bottles, and, more recently, with the use of laptops [2-3]. EAI is also considered by some as a behavioral disturbance and seen more commonly in patients with mental health issues and low intelligence quotient [4]. 

Patients suffering from chronic pain, such as our patient, are often at a higher risk than the general population [5-6]. Similarly, in patients undergoing rehabilitation, EAI can develop after receiving heat therapy for pain and inflammation [7]. It is also frequently found in temperate countries where the use of a variety of heat sources is widespread in the winter [7].

The exact pathophysiology of the condition is unknown. It is hypothesized that the prolonged heating exposure or exposure to the infrared radiation, but below the threshold, causes burns leading to the skin changes. With this extended and repeated heat exposure, damage to the superficial blood vessels leads to hemosiderin deposition. Over time, it leads to the development of hyperpigmentation, hyperkeratosis, as well as hyper-elastosis of the exposed skin [8-9].

The histopathologic findings at the microscopic level can vary from epidermal thickening and hyperpigmentation to necrosis. Melanin and hemosiderin deposits in the dermis, as well as the presence of perivascular infiltrate, is also typically seen [10]. There could be an accumulation of dermal elastic tissue [11]. Cases have also been reported with findings similar to actinic keratoses [12-13].

The distribution on the skin is affected by the source of heat, the direction of the incident radiation, the skin type, and the interposing clothing [14-15]. As mentioned earlier, it is a regional skin involvement with reticular and hyperpigmentation secondary to mild heat in the range of 43-47 degrees centigrade [2]. Prolonged heat exposure can lead to atrophy, keratosis, or even bullae formation. There have been case reports of an increased association of bullous EAI in diabetic patients, but further research is needed to prove this association [16].

Similar presenting conditions like vasculitis, livedo reticularis [14], cutis marmorata, poikiloderma, systemic lupus erythematosus, antiphospholipid syndrome and Sneddon’s syndrome [17] should be part of the differential diagnosis for EAI. Hyperpigmentation can be seen in conditions like stasis dermatitis, post-inflammatory changes, and repeated stimulus application that can mimic lichen simplex chronicus [17]. It is important that we advise patients to remove the source of heat. The lesions typically clear in weeks to months on their own after removal of the offending agent. In the case of persistent symptoms, laser therapy and tretinoin can be tried [12,17].

Cases of epidermal atypia to full-blown Merkel cell carcinoma and squamous cell carcinoma have been observed with long-standing EAI [13,18]. Biopsies should be performed for severe, persistent, and non-healing sores to rule out the development of ominous changes such as squamous cell carcinoma. It has been reported in patients with internal malignancy but is not a marker of internal malignancy [19].

## Conclusions

We, as providers, must be aware of EAI, which might be making a comeback, especially in the high-risk population we discussed above. We should also be able to differentiate it from states that closely mimic it (mentioned above). It is also essential that we educate our patients about how to prevent this condition if they are exposed to predisposing factors. Finally, clinicians should be aware of the high likelihood of skin cancers like squamous cell carcinoma developing in these lesions secondary to chronic damage. 
